# Rare-Cell Enrichment by a Rapid, Label-Free, Ultrasonic Isopycnic Technique for Medical Diagnostics**

**DOI:** 10.1002/anie.201310401

**Published:** 2014-03-26

**Authors:** Yannyk Bourquin, Abeer Syed, Julien Reboud, Lisa C Ranford-Cartwright, Michael P Barrett, Jonathan M Cooper

**Affiliations:** Division of Biomedical EngineeringUniversity of Glasgow, Glasgow, G12 8LT (UK); Institute of InfectionImmunity and Inflammation and Wellcome Trust Centre for Molecular Parasitology, University of Glasgow, Glasgow, G12 8TA (UK)

**Keywords:** diagnostic, isopycnic gradient, malaria, surface acoustic waves

## Abstract

One significant challenge in medical diagnostics lies in the development of label-free methods to separate different cells within complex biological samples. Here we demonstrate a generic, low-power ultrasonic separation technique, able to enrich different cell types based upon their physical properties. For malaria, we differentiate between infected and non-infected red blood cells in a fingerprick-sized drop of blood. We are able to achieve an enrichment of circulating cells infected by the ring stage of the parasite over nonparasitized red blood cells by between two and three orders of magnitude in less than 3 seconds (enabling detection at parasitemia levels as low as 0.0005 %). In a second example, we also show that our methods can be used to enrich different cell types, concentrating *Trypanosoma* in blood at very low levels of infection, on disposable, low-cost chips.

Parasitic infections continue to exert a disproportionate burden on the world’s poorest people. Hundreds of millions of people are infected with parasites that cause diseases like malaria and trypanosomiasis (HAT). In many cases of HAT there are fewer than 100 parasites mL^−1^ of blood, and serological and molecular tests are not considered of sufficient efficacy to offer positive diagnosis.^1^ Thus enriching these parasites from the blood of individuals with very low parasitemias will be important, if the declared intention of the World Health Organization to eliminate such diseases by 2030 is to be reached.^2^

In general, existing techniques available for enrichment of samples—whether for the identification of infectious diseases or for other acute or chronic pathologies—either exploit differences in mechanical properties, in immunologic targets, or in other affinity-based receptor–ligand interactions. Some of these methods, such as centrifugation, rely on the innate physical properties of the cell (such as density) to provide the mechanism of separation. Others, however, such as magnetic-activated cell sorting or fluorescent-activated cell sorting, require a labeling strategy.^3^ Separation techniques for diagnosis in resource-poor environments are difficult to implement particularly when complex chemical reactions are required for labeling, or where the cost of the instrumentation or the infrastructure required is high.^4^ As such, the development of a low-cost, low-power, label-free technology, which can be used with fingerprick blood sample, is paramount.

To achieve this, we have established an innovative technique based on an acoustically actuated microchip (Figure 1 a). The technique has been implemented in hand-held instrumentation which can be battery powered. In a first example, we study the performance of the microchip by enriching the circulating ring stage of *Plasmodium falciparum* in malaria-infected red blood cells (iRBCs). It is noteworthy that it is only the ring stage of this *Plasmodium* species that circulates in the peripheral blood, and is thus available within a fingerprick of blood; It is therefore important that any diagnostic or enrichment technique can demonstrate the separation of this specific parasite stage. Our method is based on developing unique flow patterns generated by the interaction of surface acoustic waves (SAWs) with blood, which, when coupled with a density gradient akin to that used in isopycnic techniques, results in the separation of parasites and parasite-infected cells from non-infected cells (Figure 1 b,c and Video 1 in the Supporting Information). Only a few microliters of blood are required, without the need for tubes, pumps, or centrifuges. Current standard methods are discussed in Note 1 in the Supporting Information

**Figure 1:**
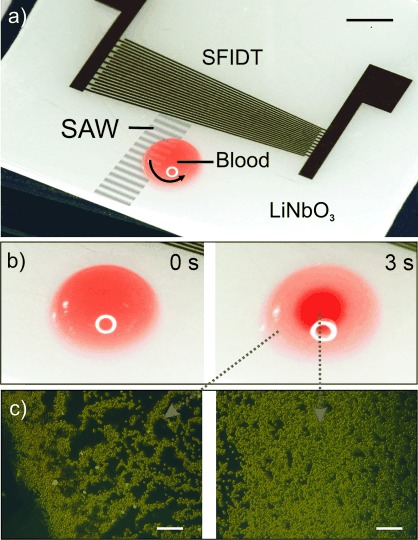
a) Photograph of the device comprising a slanted-finger interdigitated electrode (SFIDT) on LiNbO_3_. The surface acoustic wave (SAW) is generated at a defined position asymmetrically with respect to the drop of blood, thereby inducing a rotational motion within the drop. Scale bar: 3 mm. b) Photographs of a droplet of blood before (left) and after (right) actuation with SAW (3 s). In the actuated droplet, the red blood cells (RBCs) are concentrated in the middle, while infected RBCs (iRBCs) are enriched at the periphery. Scale bars: 1.5 mm. c) Fluorescent micrographs of the enriched iRBCs at the periphery (left) and concentrated RBCs in the center of the drop (right). Acridine orange (1.5 μg mL^−1^) was added to the solution to stain the parasites. Scale bars: 100 μm.

More challenging is the diagnosis of sleeping sickness or HAT, which is a deadly parasitic disease endemic in sub-Saharan Africa, and where the detection of parasite levels of 100 to 10 000 trypanosomes mL^−1^ of blood is necessary (the limit of detection using a thick blood smear only reaches 5000 trypanosomes mL^−1^ 5). Immunodiagnostics or traditional stains are not effective for the large number of patients with low parasitemia, who are consequently neither diagnosed nor treated.^6^ Identifying and treating those patients with subclinical parasite burdens is key to achieving the elimination of HAT.^2^ Realizing the potential to bring such diagnostic assays away from centralized laboratories to point-of-care settings and implementing tests to resource-limited settings will underpin future disease control strategies.^7^

Here, we describe a method that involves actuating fluid on a low-cost, disposable chip by means of a SAW.^8^ When a drop of blood is placed in the path of this SAW, the mechanical waves refract, generating fluid streaming.^8^, ^9^ Important applications have already emerged from such acoustic technologies in the field of diagnostics such as SAW-based immunoassays^10^ and nucleic acid amplification.^11^ We now demonstrate a step change in diagnostic sample processing, providing a new suitable format for the ultrasensitive analysis of blood from a fingerprick sample.

The technique relies upon controlling the shape of the acoustic field to generate a unique pattern of fluid streaming within the blood. Asymmetric actuation of the drop on the surface of the piezoelectric substrate with the SAW (Figure 1 a) causes a circular rotational motion, inducing secondary flows (Figure 2 a) in a manner similar to Batchelor flows^12^–^15^ (see Note 2 in the Supporting Information). Here we have used a slanted finger interdigitated electrode (SFIDT; Figure 1 a) to create a narrow path of propagation, providing the asymmetry in the propagation of the waves.^16^

**Figure 2:**
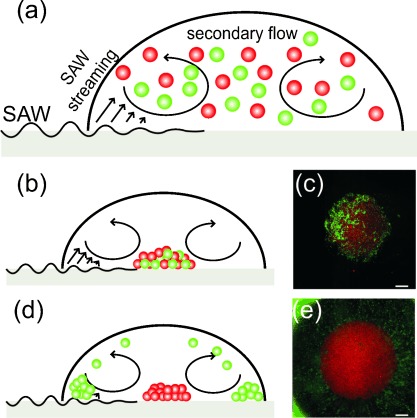
Characterization of the separation mechanism using 5 μm fluorescent beads: silica (red, *ρ*=2 g cm^−3^) and polystyrene (green, *ρ*=1.05 g cm^−3^). a) Flows induced by the SAW within the drop at the beginning of the process, when the beads are distributed in the liquid. b) After actuation, for *ρ*_f_=1 g cm^−3^, both types of beads were concentrated in the center of the drop as shown in the micrograph (c). d) For *ρ*_f_=1.160 g cm^−3^, the lighter polystyrene beads were distributed at the periphery (e). Scale bars: 300 μm.

A model system consisting of fluorescent microbeads of two different densities was first used to characterize the method. This showed clear separation between the dense beads, which accumulated in the middle of the drop and the less-dense beads, which were carried to the periphery (Figure 2 c,e). In detail, when the density of the sample matrix was lower than that of both types of beads, all the beads collected in the center of the drop (Figure 2 b,c). However, when the density of the sample matrix was lower than that of the red beads but higher than that of the green beads, the buoyancy (F_B_) and the drag force (F_D_) of the green beads was were sufficiently strong to overcome gravity (F_g_), lifting only the green beads into the flow field (Figure 2 d,e and Note 3 in the Supporting Information). As a consequence, the more dense red beads (Figure 3) were accumulated in the center of the droplet whilst the lighter green beads were enriched at the periphery. The enriched material was then easily isolated and analyzed.

**Figure 3:**
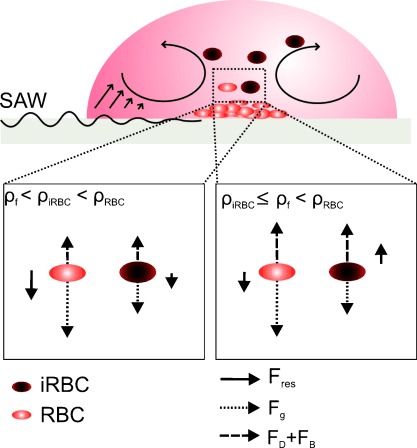
Diagram of the flow pattern within the droplet. The SAW induces streaming in the drop and a secondary flow brings the particles to the center of the drop. The two lower diagrams show the forces acting on the RBCs and iRBCs for different values of *ρ*_f_. When *ρ*_iRBC_≤*ρ*_f_<*ρ*_RBC_, the buoyancy (F_B_) and drag force (F_D_) overcome gravity (F_g_) and lift the iRBCs but not the RBCs.

It is already established that infection of RBCs by *P. falciparum* causes changes in the physical properties of RBCs, altering their surface biochemistry, elasticity, volume, and density.^17^ The densities of RBCs infected with *P. falciparum* (1.077–1.080 g cm^−3^) are lower than those of uninfected RBCs (1.080–1.110 g cm^−3^).^18^, ^19^ The enrichment system was operated using parasite-infected RBCs (iRBCs), initially containing a mixture of the different asexual stages of the parasite. While the enrichment of the infected blood cells was not dependent on the frequency of the SAW (Figure S1 in the Supporting Information), we showed that there was an optimum input power (between 65 to 250 mW) for densities of the sample above 1.083 g cm^−3^ (Figure 4 a,b). Under these conditions, a critical fluid flow velocity was achieved which generated sufficient drag force to lift the iRBCs into the flow patterns shown in Figure 3. Stronger streams generated at powers above 250 mW also lifted uninfected RBCs, negating the enrichment process (see Figure 4 b).

**Figure 4:**
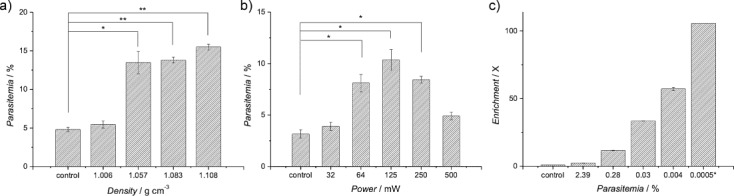
a) The acoustic enrichment of *P. falciparum* ring-stage parasites as a function of the density of the solution (at 10 MHz, 100 mW, 3 s). Controls indicate the parasitemia in the sample used prior to microseparation. Parasitemias were determined by Giemsa staining. The data are means of nine replicates and the error bars are standard errors of the mean. Statistically significant enrichments are marked with * (95 % confidence) and ** (99 %). b) The effect of the input power on the enrichment of *P. falciparum* for a frequency of 10 MHz and solution density of 1.083 g cm^−3^. c) Enrichment (ratio of parasitemia after processing, over initial parasitemia) of ring-stage malaria parasites at parasitemias from 2.39 % to 0.0005 % in Histodenz at 15 % (at 10 MHz, 100 mW, 3 s). The control for 0.0005 % parasitemia was determined by serial dilutions.

As only the ring stage of the parasite is detectable in the circulating blood, we further demonstrated its enrichment at a frequency of 10 MHz, a power of 100 mW, and an optimal density of the solution above 1.083 g cm^−3^ (Figure 4 a). The limit of the effectiveness of the system was investigated for low parasitemias and it was found that as the parasitemia decreased to 0.0005 % (25 parasites μL^−1^), the enrichment increased to 100-fold (Figure 4 c). The enriched material could be detected microscopically on a thin smear, which would not have been possible using the blood before enrichment.

This method of enrichment relies on the contrasting mechanical properties of the different cell types present in the sample. As such, it has the potential to be used for a wider range of diseases. To illustrate the broader applicability, we showed that members of the *Trypanosoma* genus of protozoa can also be enriched from infected blood at an optimum density of 1.083 g cm^−3^ (Figure 5 a). *Trypanosoma cyclops* was enriched from blood on a low-cost platform comprising a disposable chip interfaced with the SAW actuation.^10^, ^11^ The sample was positioned on a microchip, onto which phononic structures had been manufactured.^13^ The phononic lattices provided the capability to filter the acoustic waves, creating the asymmetric flow required for the enrichment process. In this case, we used a conventional “straight” interdigitated electrode (IDT) to generate the acoustic actuation (see Methods and Figure S2 in the Supporting Information). The disposable chip, which can be fabricated using low-cost materials (e.g. glass), is coupled onto the reusable piezoelectric actuator. Figure 5 b shows an enrichment factor over 100-fold with a parasitemia of 4.3×10^−5^ % (or ca. 6 trypanosomes mL^−1^), again allowing detection of parasites at a level not possible using current techniques in isolation. The results (Figure 5 b) show enrichment comparable to that obtained on the piezoelectric substrate (“slanted IDT”).

**Figure 5:**
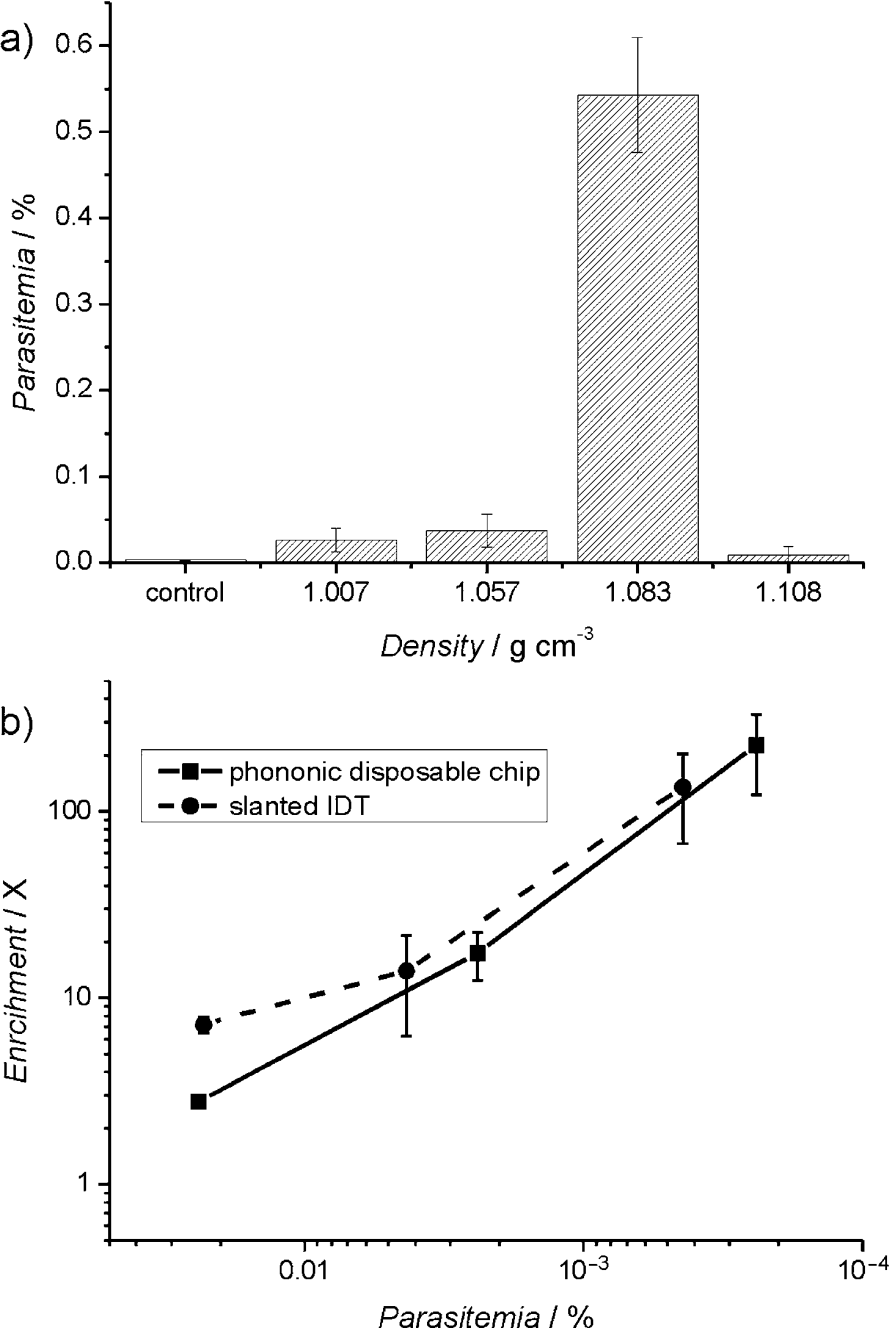
a) The concentration effect of SAW microseparation on *Trypanosoma cyclops* suspended in Histodenz as a function of the density of the solution for a frequency of 10.9 MHz. b) Enrichment achieved following SAW microseparation on a slanted IDT and a disposable superstrate (8.8 MHz, 1 W, 5 s) of samples containing trypanosomes in 15 % Histodenz. Parasitemias were determined by hemocytometry in samples taken from the periphery of the droplet following SAW microseparation. The data are means of three replicates; the error bars are standard errors of the mean.

In conclusion, the use of SAW as described here provides a low-power, low-cost, label-free technique appropriate for resource-limited settings, which could find broad application in both point-of-care and field settings. The power required for enrichment indicates that over 30 000 sample manipulations could be run from the charge of a mobile phone when the test is performed directly on the piezoelectric actuator, and 2000 assays when a disposable device is used. Most simply, in a field setting, the enriched samples could be stained (Giemsa or acridine orange) and examined microscopically, decreasing the limit of detection of the current gold standard for malaria and sleeping sickness by more than two orders of magnitude. Our methods could also work alongside an LED-based field fluorescence microscope^20^ or a lensfree system, providing both enrichment and detection within the same device. We could also use this method in the diagnosis of other infectious diseases, as well as in the study of circulating tumor cells, which have also been reported to have different mechanical properties.^21^
