# Supplementary material for: Rare-Cell Enrichment by a Rapid, Label-Free, Ultrasonic Isopycnic Technique for Medical Diagnostics
Source: Angew Chem Int Ed Engl. 2014 Mar 26;53(22):5587–90. doi: 10.1002/anie.201310401 (PMC4232275; doi:10.1002/anie.201310401)
Supplement: Supplementary file 1 [file anie0053-5587-sd1.pdf]

Angewandte Chemie
Eine Zeitschrift der Gesellschaft Deutscher Chemiker

Supporting Information
© Wiley-VCH 2014
69451 Weinheim, Germany

Rare-Cell Enrichment by a Rapid, Label-Free, Ultrasonic Isopycnic Technique for Medical Diagnostics**

*Yannyk Bourquin, Abeer Syed, Julien Reboud, Lisa C. Ranford-Cartwright, Michael P. Barrett, and Jonathan M. Cooper**

anie_201310401_sm_miscellaneous_information.pdf
anie_201310401_sm_movie_centrifuge.avi

Supporting Information

Experimental Section

Device Fabrication. The SAW devices were fabricated on a 128o Y-cut X-propagating 3 inch $LiNbO_3$ wafer. The slanted finger interdigitated transducer (SFIDT) consisted of 10 pairs of fingers. The width of the tapered fingers and the space between them varied linearly from 62.5 µm to 125 µm along the aperture1. The straight IDT, used as an actuator for the phononic microchip, was based on a previously published design,[11] yielding an operating frequency around 9 MHz. The $LiNbO_3$ wafer was coated by spinning AZ4562 photoresist before transferring the pattern using standard photolithography and developed using AZ400K. A 20 nm titanium adhesion layer was evaporated prior to deposition of 100 nm of gold and lift-off was then performed in acetone. For the observation of fluorescent beads and parasites stained with acridine orange, the electrodes where patterned on a transparent double side polished $LiNbO_3$ wafer.

The protocols for the fabrication of such phononic device has been described previously.[11] This structure comprised a square array (pitch 203 µm) of circular holes (radius 82 µm), dry-etched (STS) in a 470 µm-thick [100] silicon wafer. A hydrophilic spot was created on the surface of the devices to reproducibly position the blood. The devices were coated with AZ4562 photoresist, patterned using standard photolithography and developed using AZ400K. The pattern comprised a disk of 3 mm diameter. The devices were then treated in O2 Plasma for 2 min at 100 W before salinization in a solution of 30 µl of trichloro(1H,1H,2H,2H-perfluorooctyl)silane (Aldrich) in 50 ml of heptane for 10 min. The photoresist was removed in acetone and the surface was rinsed with methanol and blow dried. The SAW device was finally positioned on a heat sink.

The SAW device was characterized using a Network Analyzer (Agilent Technologies E5071C ENA series) and a vibrometer (Polytec). The SFIDT was connected to a TGR1040 - 1GHz RF Generator (Thurlby Thandar Instruments) in conjunction with a Mini Circuits ZHL-5W-1, 5 – 500 MHz amplifier and a 3A, 24V dc power supply to generate the SAW.

Density solutions. The gradient solutions used were Optiprep (Sigma-Aldrich) and Histodenz (Sigma-Aldrich). For experiments with beads, Optiprep was mixed with deionized water to a concentration of 30 %. For the experiments with infected red blood cells, Histodenz was diluted in deionized water to a concentration of 30 % in order to have a high density solution with osmolarity of 300 mOsm. This solution was then further diluted in the culture medium. The density of the solution was measured using DA-510 (Kyoto Electronics Manufacturing) and the osmolarity was determined using The Advanced Micro Osmometer Model no. 3300 (Advanced Instruments Inc). The density for Optiprep 30 % was 1.160 g cm^{-3}, while Histodenz 0, 10, 15, and 20 % in RPMI were 1.006, 1.057, 1.083, 1.108 g cm^{-3} respectively.

iRBCs preparation *P. falciparum* clone 3D7 was grown in vitro according to standard protocols using human erythrocytes obtained from the Scottish National Blood Transfusion Service. Parasites were tightly synchronized to obtain ring-stage parasites.[S1] Parasite samples for SAW analysis were pelleted by centrifugation (400 g for 5 minutes) and resuspended in culture medium (RPMI1640 with 10 % human serum) at 40 % haematocrit, to mimic that seen in human blood. Thin blood smears were prepared and stained with 5 % Giemsa's stain for 40 minutes and examined under 1000 x magnification (oil immersion), with a minimum of 100 parasites counted to obtain an accurate estimate of parasitaemia. Serial dilutions were performed using uninfected human blood in culture medium (RPMI1640 with 10 % human serum) as the diluent, from that in the culture (2.4 %) down to a final parasitaemia of 0.0005 %.

Trypanosoma cyclops preparation. *T. cyclops* trypomastigotes have proven a suitable surrogate for *T. brucei* in microfluidic studies of these parasites.[S2] They were cultured in Cunningham's medium supplemented with 20 % FBS and maintained at 27 °C. For the use in experiments, 1 mL of trypanosomes in culture medium was centrifuged at 1000 g for 5 minutes and the pellet re-suspended in fresh medium. Human blood was spiked with 10,000 trypanosomes mL^{-1} to mimic the human parasitaemia levels (0.00237 %). For $4.4*10^{-4}$ and $4.32*10^{-4}$ %, the blood was spiked with 1000 and 100 trypanosomes mL^{-1} respectively.

iRBCs enrichment. The infected blood was mixed with the density gradient solutions to a final percentage of RBCs of ~2 % in each experiment. A finger prick volume of blood (10 µl) was then pipetted on to the SAW device. The SAW was actuated for 3 seconds for the separation and the outer part of the droplet was pipetted out. A thin smear was then created from this solution and stained using 5 % Giemsa staining for 40 minutes. The number of parasites was counted under microscope as above.

For the experiments with fluorescence detection, 10 µl of acridine orange (stock solution of 0.15 mg ml^{-1}) was added to 1 ml of infected blood before the SAW process and the resulting solution was directly observed under a fluorescent microscope (Ex 480 nm, Em 535 nm).

Trypanosoma enrichment. The spiked blood was mixed with the density gradient solutions to a final percentage of RBCs of ~1 % in each experiment. A fingerprick volume of blood (10 µl) was then pipetted on to the SAW device. The SAW was actuated for 5 seconds for the separation and the outer part of the droplet was pipetted out. The samples were then analysed using haemocytometry.

Supplementary Note 1 – Gold standard methods for Malaria detection

Current "gold standard" for malaria diagnosis still relies on direct observation of the parasite in a Giemsa-stained blood smear using an optical microscope. This approach requires a laboratory setting, specialized technicians and is time consuming (~60 min) – with a limit of detection using a thin blood smear around 100 parasites μl^{-1} and 5-20 parasites μl^{-1} for a thick blood smear.[S3] Rapid tests based upon immunodiagnostics involving antigen capture have been used in endemic areas where microscopy is not available, although such techniques are not sufficiently reliable to detect low parasitaemias (below 200 parasites μl^{-1}).[S4]

Supplementary Note 2 – Batchelor Flows

Batchelor flows are described as occurring in a cylindrical column of fluid with a rotating disk on the top of the drop and a stationary one at the bottom, where the column interfaces with the piezoelectric diagnostic chip. This interface between the chip and the droplet is a no-slip boundary (equivalent to a stationary disk) whilst the top surface of the droplet can be seen as a surface with the largest azimuthal velocity due to absence of friction with air (as stated, equivalent to the rotating disk). As a consequence of frictional effects at the surface of the chip, the azimuthal velocity is smaller at the bottom than at the top of the droplet, causing the fluid to travel inward at the interface with the piezoelectric surface and drawing the RBCs to the centre of the drop. Constraints arising from the capillarity and volume conservation results in the fluid recirculating

upward in the middle of the droplet and downward at the free surface as illustrated in Figure 2. We use an isopycnic gradient, adjusting the density of the fluid (ρ_f) by adding agents such as Histodenz™ so that it becomes higher than that of the parasites (ρ_p) or parasite-infected cells (ρ_{iRBC}) but lower than that of the RBCs (ρ_{RBC}).

Supplementary Note 3 – Forces

The drag force on a particle, directed upward, can be estimated by:

$$F_D = C_D 12 \mu U a \qquad [1]$$

where C_D is the drag coefficient, μ is the dynamic viscosity, U is the velocity of the fluid and a is the equivalent radius of the particle (for RBCs, $a = (3V / 4p)^{1/3}$, where V is the volume of the RBC).[13] The buoyancy also directed upward can be estimated by:

$$F_B = \frac{4}{3}\pi a^3 \rho_f g \qquad [2]$$

where g is the gravitational acceleration. The force due to the gravity is directed downward and is estimated by:

$$F_g = \frac{4}{3}\pi a^3 \rho_p g \qquad [3]$$

Current "gold standard" for malaria diagnosis still relies on direct observation of the parasite in a Giemsa-stained blood smear using an optical microscope. This approach requires a laboratory setting, specialized technicians and is time consuming (60 min) – with a limit of detection using a thin blood smear around 100 parasites μl^{-1} and 5-20 parasites μl^{-1} for a thick blood smear.[6] Rapid tests based upon immunodiagnostics involving antigen capture have been used in endemic areas where microscopy is not available, although such techniques are not sufficiently reliable to detect low parasitaemias (below 200 parasites μl^{-1}).[7]

Additional References.

[S1] L. C. Ranford-Cartwright, A. Sinha, G. S. Humphreys, J. M. Mwangi, *Malar*. J. **2010**, 9, 170.

[S2] S. H. Holm, J. P. Beech, M. P. Barrett, J. O. Tegenfeldt, *Lab. Chip* **2011**, 11, 1326–1332.

[S3] L. Ochola, P. Vounatsou, T. Smith, M. Mabaso, C. Newton, *Lancet Infect. Dis.* **2006**, *6*, 582–588.

[S4] M. L. McMorrow, M. Aidoo, S. P. Kachur, *Clin. Microbiol. Infect.* **2011**, *17*, 1624–1631.

Supplementary Movie.

Movie M1. Separation of iRBCs from RBCs. (See Figure 1a-b in the main text for details). The surface acoustic wave (SAW) is generated at a defined position asymmetrically with respect to the drop of blood, thereby inducing a rotational motion within the drop. After 3 s, the more dense red blood cells form a 'pellet' in the center of the drop, while the lighter infected red blood cells are enriched at the periphery. A bright ring can be seen within the drop, a reflection from the illumination.

Supplementary Figure S1

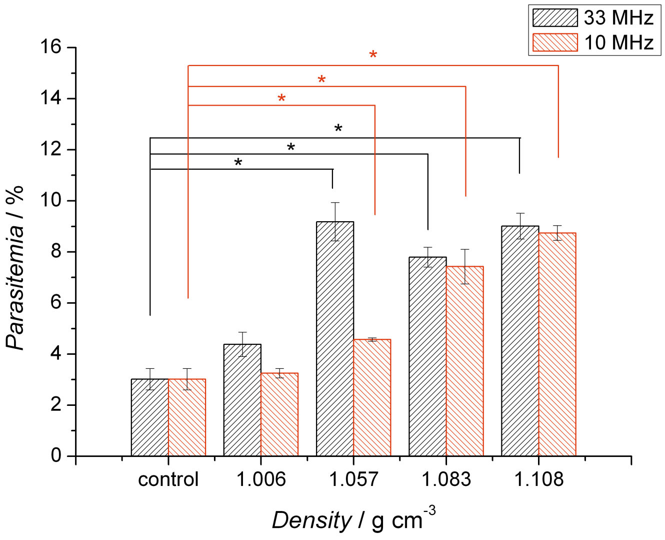

Figure S1. (a) The concentration effect of SAW microseparation on *P. falciparum* parasites suspended in Histodenz as a function of the density of the solution (SAW input frequency of 10 MHz and 33 MHz, power of 100 mW). Control indicates the parasitaemia in the sample used prior to SAW microseparation, and the other points are the parasitaemia recorded in samples taken from the periphery of the droplet following SAW microseparation. The data are means of 9 replicates with standard errors of the mean. Statistically significant enrichments are marked with * (95 % confidence).

Supplementary Figure S2

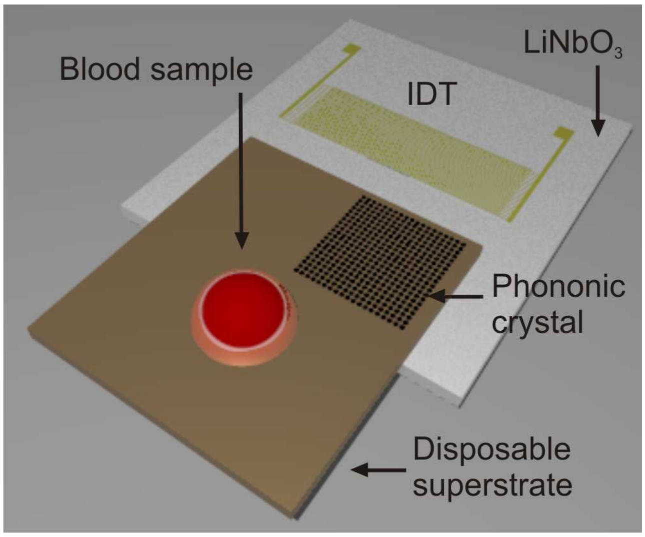

Figure S2. Schematic of the SAW arrangement using a disposable superstrate, coupled to the $LiNbO_3$ IDT by a water-based gel, as described in reference 13. The waves generated by the IDT propagate as Lamb waves in the superstrate and are shaped by the phononic crystal structures to induce the rotational momentum leading to separation.
